# The Hyaluronic Acid–HDAC3–miRNA Network in Allergic Inflammation

**DOI:** 10.3389/fimmu.2015.00210

**Published:** 2015-04-30

**Authors:** Youngmi Kim, Sangkyung Eom, Deokbum Park, Hyuna Kim, Dooil Jeoung

**Affiliations:** ^1^Department of Biochemistry, College of Natural Sciences, Kangwon National University, Chuncheon, South Korea

**Keywords:** allergic inflammation, CD44, histone deacetylase-3, hyaluronic acid, micro RNA genes

## Abstract

We previously reported the anti-allergic effect of high molecular weight form of hyaluronic acid (HMW-HA). In doing so, HA targets CD44 and inhibits FcεRI signaling and cross-talk between epidermal growth factor receptor (EGFR) and FcεRI. We previously reported the role of histone deacetylases (HDACs) in allergic inflammation and allergic inflammation-promoted enhanced tumorigenic potential. We reported regulatory role of HA in the expression of HDAC3. In this review, we will discuss molecular mechanisms associated with anti-allergic effect of HA in relation with HDACs. The role of microRNAs (miRNAs) in allergic inflammation has been reported. We will also discuss the role of miRNAs in allergic inflammation in relation with HA-mediated anti-allergic effects.

## The Role of Hyaluronic Acid in Allergic Inflammation

Hyaluronic acid (HA), a major component of the extracellular matrix (ECM), plays a key role in regulating inflammation. HA enhances proteoglycan synthesis, reduces the production and activity of pro-inflammatory mediators and matrix metalloproteinases, and alters the behavior of immune cells (1). Inflammation is associated with accumulation and turnover of HA polymers by multiple cell types. Increased accumulation of HA has been demonstrated in joint tissue of rheumatoid arthritis (RA) patients (2); in lung disease, both in humans (3) and animal experimental models (4); in inflammatory liver disease; during vascular disease (5); in rejected kidney transplants (6) as well renal tissue of patients experiencing diabetic nephropathy (7); in the intestine of patients undergoing flares of inflammatory bowel disease (IBD) (8).

Circulating HA might be a marker of asthma control, as it correlates with airway resistance and has good sensitivity in the detection of impaired asthma control (9). The increased level of HA is correlated with asthma (10). In addition, HA appears to provide the scaffolding for inflammatory cell accumulation as well as for new collagen synthesis and deposition (10). HA deposition appears largely due to up-regulation of hyaluronan synthase 1 (HAS1) and hyaluronan synthase 2 (HAS2). HAS2 mRNA is markedly increased in asthmatic fibroblasts (11). In cases of inflammation, HA contains a variety of HA polymers with overlapping lengths and functions. HA exists as both a pro-and anti-inflammatory molecule *in vivo*, and these contradictory functions depend upon polymer length. High molecular weight form of hyaluronic acid (HMW-HA) elicits protective anti-inflammatory effects that protect lung epithelial cells from apoptosis and is protective against liver injury, acting to reduce pro-inflammatory cytokines in a T-cell-mediated injury model (12). HMW-HA inhibits macrophage proliferation and cytokine release, leading to decreased inflammation in the early wound of a preclinical post laminectomy rat model (13). HMW-HA exerts a negative effect on the activation of mitogen-activated protein kinase (MAPK) by allergic inflammation (14). HA with an average molecular mass <500 kDa can be considered a fragment. HA fragments with an average molecular weight of 200 kDa have been shown to stimulate chemokines, cytokines, growth factors, proteases, and by macrophages (15–20). Organic contact sensitizers induce production of reactive oxygen species (ROS) and a concomitant breakdown of HA to pro-inflammatory low molecular weight fragments in the skin (21). Importantly, inhibition of either ROS-mediated or enzymatic HA breakdown prevents sensitization as well as elicitation of Chediak–Higashi Syndrome (CHS) (21). Mucus hyper secretion with elevated MUC5B mucin production is a pathologic feature in many airway diseases associated with oxidative stress (22). ROS-induced MUC5AC expression in normal human bronchial epithelial cells (NHBE) is dependent on HA depolymerization and epidermal growth factor receptor (EGFR)/MAPK activation (22). Although most of the work on low molecular weight HA (LMW-HA) fragments initially illustrated a pro-inflammatory response, a number of studies have shown that HA fragments can also be protective. In a murine model of colitis, intraperitoneal injection of HA <750 kDa protects colonic epithelium in a Toll-like receptor (TLR) 4-dependent manner (23). This functional difference between HAs of varying sizes is a matter of controversy since many studies have reported opposing results in regard to which type of HA can bring about cellular changes (24). These contradictory functions of HA, depending on the polymer length, may result from differential effects of these HA on HA receptors such as CD44 and receptor for HA-mediated motility (RHAMM). Exogenous HAs used in many studies are not homogenous with respect to size. Therefore, it is difficult to conclude that size alone determines the function of HAs of various sizes. These discrepancies may also be due to differences in experimental settings, purity of HA (25), and the possibility of diverse responses to HA depending on the cell type. Although many reports suggest anti-allergic effect of exogenous HA, the effect of endogenous HMW-HA on the allergic inflammation needs further investigation.

Hyaluronic acid levels are elevated in allergic animals and the increase correlates with the influx of inflammatory cells. This increase in HA levels is largely due to up-regulation of hyaluronidase-1 (HYAL-1) and hyaluronidase-2 (HYAL-2) (26). HYAL-1, -2, and -3 are expressed in airway epithelium and may operate in a coordinated fashion to depolymerize HA during allergen-induced asthmatic responses associated with up-regulation of tumor necrosis factor-alpha (TNF-alpha) and interleukin-1 beta (IL-1beta) (27). Degradation of HA by HYAL-1 primarily depends upon CD44 or other HA receptors to internalize HA fragments. Patients deficient in HYAL-1 have been reported with plasma HA levels at 40 times normal (28). The finding of HYAL-2 in complex with CD44 at the plasma membrane suggests that HA-binding proteins may enhance the activity of HA degrading enzymes, and CD44 binding may provide HYAL-2 with a preferable conformation of HA. IL-1beta exerts inflammatory activity via CD44 by the mediation of HA fragments derived from HA depolymerization (29).

CD44, a receptor for HA, expressed on CD4(+) T cells plays a critical role in the accumulation of antigen-specific Th2 cells, but not Th1 cells, in the airway and in the development of airway hyper-responsiveness (AHR) induced by antigen challenge (30). Airway fibroblasts from patients with asthma produced significantly increased concentrations of LMW-HA compared with those of normal fibroblasts (30). CD44, but not CD62L, is required for leukocyte extravasations during a Th2-type inflammatory response such as allergic dermatitis (31). HMW-HA inhibits interaction between IgE and FcεRI and between FcεRI and protein kinase C δ (PKCδ) during allergic inflammation (14). A role for CD44 in the regulation of allergic inflammation *in vivo* has been shown by studies in which anti-CD44 treatment inhibited the development of optimal contact allergic responses (32). CD44 has been shown to be responsible for the development of pulmonary eosinophilia (33). CD44-hyaluronan interaction is necessary for allergic asthma (34). The serum-derived hyaluronan-associated protein (SHAP)–HA complex has an inhibitory role in the development of airway hyper responsiveness and allergic airway inflammation which may be attributed, at least in part, to negative feedback mechanisms exerted by SHAP (35). It will be necessary to examine effects of HAs of various sizes on the expression and/or activity of CD44.

## The Role of HDAC3 in Allergic Inflammation

Histone acetylation/deacetylation plays an important role in the regulation of inflammatory genes associated with allergic inflammation (36). Histone deacetylase-3 (HDAC3)-deficient macrophages are unable to activate almost half of the inflammatory gene expression program when stimulated with lipopolysaccharide (LPS) (37). Pulmonary inflammation is ameliorated in mice lacking HDAC3 in macrophages (38). The induction of cyclooxygenase (COX)-2, which occurs during allergic inflammation, is accompanied by degradation of HDAC1 (39). HDAC2 expression and activity are decreased in asthmatic subjects, smokers, and smoking asthmatic subjects (40). HDAC3, induced by antigen stimulation, interacts with FcεRI and is necessary for allergic inflammation both *in vitro* and *in vivo* (41). DNA methyl transferase I (DNMT1) acts as a negative regulator of allergic inflammation and the down-regulation of DNMT1 induces the expression of HDAC3 (42). HDAC3 is necessary for the induction of TNF-α, a cytokine increased during allergic inflammation, in cardiomyocytes during LPS stimulation (43). HDAC3 mediates allergic inflammation by regulating the expression of monocyte chemoattractant protein-1 (MCP1) (41). HMW-HA, but not LMW-HASs, decreases the expression of HDAC3 in human vascular endothelial cells to promote angiogenesis which is accompanied by allergic inflammation (44).

## Role of miRNAs in Allergic Inflammation

**Figure 1:**
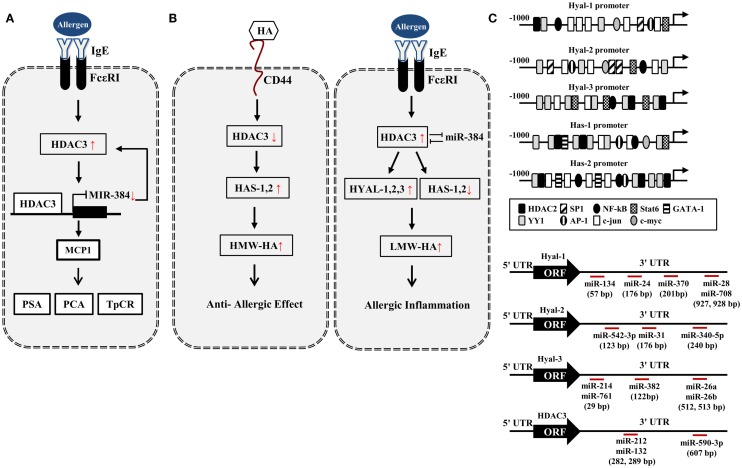
**HA–HDAC3–miRNA network in allergic inflammation**. **(A)** Allergen activates FcεRI signaling and induces the expression of HDAC3. HDAC3, through forming a negative feedback loop with miR-384, regulates allergic inflammations *in vitro* and *in vivo*. PCA, passive cutaneous anaphylaxis; PSA, passive systemic anaphylaxis; TpCR, triphasic cutaneous reaction. **(B)** Potential effect of HA metabolism on allergic inflammation. HA fragments generated by HYALs may promote allergic inflammation while high MW-HA exerts anti-allergic inflammation by HASs. **(C)** Promoter analysis shows the binding of various transcriptional regulators to the promoter sequences of HYALs and HASs. Various miRNAs bind to the 3′-UTR of HYALs and HDAC3. UTR denotes un-translated region.

microRNAs (miRNAs) are small (20–23 nucleotides), single-stranded non-coding RNAs that play important roles in the post-transcriptional regulation of gene expression in mammalian cells by regulating translation. Upon binding of their 5′ extremity (seed sequence encompassing nucleotides 2–7 or 2–8) with a complementary site located most of the time in the 3′ un-translated region (3′UTR) of target mRNAs, miRNAs alter gene expression by translational repression or RNA degradation (45). Because miRNAs regulate the expression of transcription factors that regulate the expression of miRNAs themselves, miRNAs form feedback loops. miR-384 and HDAC3 form a negative feedback loop to regulate allergic inflammation [(46), Figure 1A]. This suggests the involvement of miR-384 in the anti-allergic effect of HA. Several reports suggest role of HDACs in the expression regulation of miRNAs (47–50). miRNA let-7a regulates the expression of IL-13, a cytokine necessary for allergic lung disease (51). The down-regulation of miR-145 inhibits Th2 cytokine production and AHR (52). HA–CD44 interaction enhances the expression of miR-10b (53). miR-199a-3p and miR-34a miR-590-3p target CD44 (54, 55). Polymorphisms of CD44 3′UTR weaken the binding of miRNAs (55), suggesting that miRNAs regulate the expression of CD44. Given the fact that CD44 is involved in allergic inflammation, miRNAs may regulate HA-mediated anti-allergic inflammation.

## The Regulation of HA Metabolism by miRNAs and HDAC3

*In silico* screening of expression data with predicted miR-23 target sites combined with *in vivo* testing, predicts HAS2 as novel direct target of miR-23 (56). miR-23a-3p in non-senescent fibroblasts leads to the decreased HAS2-mediated HA synthesis (57). This implies that miR-23 may regulate the production of HA during allergic inflammation. Based on our previous report (44), HA–CD44 may decrease the expression of HDAC3 (Figure 1B). Promoter analysis shows that HAS1 and HAS2 contain the binding sites for YY1, STAT6, NF-kB, and HDAC2 (Figure 1C), suggesting that the production of HA is under epigenetic regulation. Because HDAC3 shows an inverse relationship with HDAC2 (41), HDAC3 may regulate the expression of HASs to mediate allergic inflammation. Many reports suggest that HASs may increase the production of HMW-HA to exert anti-allergic effects (Figure 1B). Thus, the decreased expression of HDAC3 by HA–CD44 interaction may increase the expression of HAS1 and HAS2 to exert anti-allergic effect (Figure 1B). HDAC3, increased during allergic inflammation, may regulate the expression of HYALs and HASs differentially to increase the production of LMW-HA. This may result in allergic inflammation (Figure 1B).

Promoter analysis shows that HYAL-1, -2, and -3 contain binding sites for various transcriptional regulators including HDAC2 (Figure 1C), suggesting the role of HDAC3 in the expression regulation of HYALs. TargetScan analysis predicts the binding of miRNAs, such as miR-24,-28, -134, and -370, to the 3′-UTR sequences of HYAL-1 (Figure 1C). TargetScan analysis predicts the binding of various miRNAs to the 3′-UTR sequences of HYAL-2 and HYAL-3 (Figure 1C). These miRNAs may prevent the production of HA fragments by negatively regulating the expression of these HYALs. Thus, these miRNAs may mediate allergic inflammation. TargetScan analysis predicts the binding of miR-212,-132, and -590 to the 3′-UTR of HDAC3 (Figure 1C). These miRNAs may exert anti-allergic effects by decreasing the expression of HDAC3. Taken together, miRNAs and HDAC3 may regulate allergic inflammation through their effects on HA metabolism.

## Concluding Remarks and Perspectives

In this study, we show the possible involvement of miRNAs and HDAC3 in the regulation of HA metabolism. HA–HDAC3 –miRNA network described in this review may offer valuable mechanism for HA-mediated anti-allergic effects. For better understanding of HA-mediated anti-allergic effect, it will be necessary to identify downstream targets of HA. The downstream targets of HA would be valuable for the development of anti-allergic drugs. Identification of more miRNAs that regulate allergic inflammation in relation to HA and HDAC3 will be necessary for better understanding of HA-mediated anti-allergic inflammation.

## Conflict of Interest Statement

The authors declare that the research was conducted in the absence of any commercial or financial relationships that could be construed as a potential conflict of interest.

## References

[1] Moreland LW. Intra-articular hyaluronan (hyaluronic acid) and hylans for the treatment of osteoarthritis: mechanisms of action. Arthritis Res Ther (2003) 5:54–67. 10.1186/ar855 12718745 PMC165033

[2] Dahl LB Dahl IM Engstrom-Laurent A Granath K. Concentration and molecular weight of sodium hyaluronate in synovial fluid from patients with rheumatoid arthritis and other arthropathies. Ann Rheum Dis (1985) 44:817–22. 10.1136/ard.44.12.817 4083937 PMC1001790

[3] Hallgren R Samuelsson T Laurent TC Modig J. Accumulation of hyaluronan (hyaluronic acid) in the lung in adult respiratory distress syndrome. Am Rev Respir Dis (1989) 139:682–7. 10.1164/ajrccm/139.3.682 2923370

[4] Jiang D Liang J Fan J Yu S Chen S Luo Y Regulation of lung injury and repair by toll-like receptors and hyaluronan. Nat Med (2005) 11:1173–9. 10.1038/nm1315 16244651

[5] Waldenstrom A Martinussen HJ Gerdin B Hallgren R. Accumulation of hyaluronan and tissue edema in experimental myocardial infarction. J Clin Invest (1991) 88:1622–8. 10.1172/JCI115475 1939649 PMC295686

[6] Wells AF Larsson E Tengblad A Fellstrom B Tufveson G Klareskog L The localization of hyaluronan in normal and rejected human kidneys. Transplantation (1990) 50:240–3. 10.1097/00007890-199008000-00014 2382292

[7] Lewis A Steadman R Manley P Craig K dela Motte C Hascall V Diabetic nephropathy, inflammation, hyaluronan and interstitial fibrosis. Histol Histopathol (2008) 23:731–9. 18366011 10.14670/HH-23.731

[8] dela Motte CA Hascall VC Drazba J Bandyopadhyay SK Strong SA Mononuclear leukocytes bind to specific hyaluronan structures on colon mucosal smooth muscle cells treated with polyinosinic acid: polycytidylic acid: inter alpha-trypsin inhibitor is crucial to structure and function. Am J Pathol (2003) 163:121–33 10.1016/S0002-9440(10)63636-X 12819017 PMC1868154

[9] Eszes N Toldi G Bohács A Ivancsó I Müller V Rigó J Jr Relationship of circulating hyaluronic acid levels to disease control in asthma and asthmatic pregnancy. PLoS One (2014) 9:e94678. 10.1371/journal.pone.0094678 24736408 PMC3988128

[10] Cheng G Swaidani S Sharma M Lauer ME Hascall VC Aronica MA. Correlation of hyaluronan deposition with infiltration of eosinophils and lymphocytes in a cockroach-induced murine model of asthma. Glycobiology (2013) 23:43–58. 10.1093/glycob/cws122 22917573 PMC3505010

[11] Liang J Jiang D Jung Y Xie T Ingram J Church T Role of hyaluronan and hyaluronan-binding proteins in human asthma. J Allergy Clin Immunol (2011) 128:403–11. 10.1016/j.jaci.2011.04.006 21570715 PMC3149736

[12] Nakamura K Yokohama S Yoneda M Okamoto S Tamaki Y Ito T High, but not low, molecular weight hyaluronan prevents T-cell-mediated liver injury by reducing proinflammatory cytokines in mice. J Gastroenterol (2004) 39:346–54. 10.1007/s00535-003-1301-x 15168246

[13] Schimizzi AL Massie JB Murphy M Perry A Kim CW Garfin SR High molecular-weight hyaluronan inhibits macrophage proliferation and cytokine release in the early wound of a preclinical post laminectomy rat model. Spine J (2006) 6:550–6. 10.1016/j.spinee.2005.12.005 16934726

[14] Kim Y Lee YS Hahn JH Choe J Kwon HJ Ro JY Hyaluronic acid targets CD44 and inhibits FcepsilonRI signaling involving PKCdelta, Rac1, ROS, and MAPK to exert anti-allergic effect. Mol Immunol (2008) 45:2537–47. 10.1016/j.molimm.2008.01.008 18289679

[15] Horton MR Burdick MD Strieter RM Bao C Noble PW. Regulation of hyaluronan-induced chemokine gene expression by IL-10 and IFN-gamma in mouse macrophages. J Immunol (1998) 160:3023–30. 9510207

[16] Horton MR McKee CM Bao C Liao F Farber JM Hodge-Du Four J Hyaluronan fragments synergize with interferon-gamma to induce the C-X-C chemokines mig and interferon-inducible protein-10 in mouse macrophages. J Biol Chem (1998) 273:35088–94. 10.1074/jbc.273.52.35088 9857043

[17] Horton MR Olman MA Noble PW Hyaluronan fragments induce plasminogen activator inhibitor-1and inhibit urokinase activity in mouse alveolar macrophages: a potential mechanism for impaired fibrinolytic activity in acute lung injury. Chest (1999) 116:17S. 10424564

[18] Horton MR Shapiro S Bao C Lowenstein CJ Noble PW. Induction and regulation of macrophage metalloelastase by hyaluronan fragments in mouse macrophages. J Immunol (1999) 162:4171–6. 10201943

[19] Noble PW Lake FR Henson PM Riches DW. Hyaluronate activation of CD44 induces insulin-like growthfactor-1expression by a tumor necrosis factor-alpha-dependent mechanism in murine macrophages. J Clin Invest (1993) 91:2368–77. 10.1172/JCI116469 8514850 PMC443294

[20] McKee CM Penno MB Cowman M Burdick MD Strieter RM Bao C Hyaluronan (HA) fragments induce chemokine gene expression in alveolar macrophages. The role of HA size and CD44. J Clin Invest (1996) 98:2403–13. 10.1172/JCI119054 8941660 PMC507693

[21] Esser PR Wölfle U Durr C von Loewenich FD Schempp CM Freudenberg MA Contact sensitizers induce skin inflammation via ROS production and hyaluronic acid degradation. PLoS One (2012) 7:e41340. 10.1371/journal.pone.0041340 22848468 PMC3405137

[22] Casalino-Matsuda SM Monzon ME Day AJ Forteza RM. Hyaluronan fragments/CD44 mediate oxidative stress-induced MUC5B up-regulation in airway epithelium. Am J Respir Cell Mol Biol (2009) 40:277–85. 10.1165/rcmb.2008-0073OC 18757307 PMC2645525

[23] Zheng L Riehl TE Stenson WF. Regulation of colonic epithelial repair in mice by toll-like receptors and hyaluronic acid. Gastroenterology (2009) 137:2041–51. 10.1053/j.gastro.2009.08.055 19732774 PMC2789856

[24] McKee CM Lowenstein CJ Horton MR Wu J Bao C Chin BY Hyaluronan fragments induce nitric-oxide synthase in murine macrophages through a nuclear factor kappaB-dependent mechanism. J Biol Chem (1997) 272:8013–8. 10.1074/jbc.272.12.8013 9065473

[25] McDonald JA Camenisch TD Hyaluronan: genetic insights into the complex biology of a simple polysaccharide. Glycoconj J (2003) 19:331–9 10.1023/A:1025369004783 12975613

[26] Ghosh S Samarasinghe AE Hoselton SA Dorsam GP Schuh JM. Hyaluronan deposition and co-localization with inflammatory cells and collagen in a murine model of fungal allergic asthma. Inflamm Res (2014) 63:475–84. 10.1007/s00011-014-0719-3 24519432 PMC4020973

[27] Monzón ME Manzanares D Schmid N Casalino-Matsuda SM Forteza RM. Hyaluronidase expression and activity is regulated by pro-inflammatory cytokines in human airway epithelial cells. Am J Respir Cell Mol Biol (2008) 39:289–95. 10.1165/rcmb.2007-0361OC 18390475 PMC2542447

[28] Triggs-Raine B Salo TJ Zhang H Wicklow BA Natowicz MR. Mutations in HYAL1, a member of a tandemly distributed multigene family encoding disparate hyaluronidase activities, cause a newly described lysosomal disorder, mucopolysaccharidosis IX. Proc Nat l Acad Sci U S A (1999) 96:6296–300. 10.1073/pnas.96.11.6296 10339581 PMC26875

[29] Campo GM Avenoso A D’Ascola A Scuruchi M Prestipino V Calatroni A Hyaluronan in part mediates IL-1beta-induced inflammation in mouse chondrocytes by up-regulating CD44 receptors. Gene (2012) 494:24–35. 10.1016/j.gene.2011.11.064 22192912

[30] Katoh S Kaminuma O Hiroi T Mori A Ohtomo T Maeda S CD44 is critical for airway accumulation of antigen-specific Th2, but not Th1, cells induced by antigen challenge in mice. Eur J Immunol (2011) 41:3198–207. 10.1002/eji.201141521 21874648

[31] Gonda A Gál I Szántó S Sarraj B Glant TT Hunyadi J CD44, but not l-selectin, is critically involved in leucocyte migration into the skin in a murine model of allergic dermatitis. Exp Dermatol (2005) 14:700–8. 10.1111/j.0906-6705.2005.00348.x 16098130

[32] Camp RL Scheynius A Johansson C Pure E. CD44 is necessary for optimal contact allergic responses but is not required for normal leukocyte extravasation. J Exp Med (1993) 178:497–507. 10.1084/jem.178.2.497 8340756 PMC2191099

[33] Katoh S Matsumoto N Kawakita K Tominaga A Kincade PW Matsukura S. A role for CD44 in an antigen-induced murine model of pulmonary eosinophilia. J Clin Invest (2003) 111:1563–70. 10.1172/JCI16583 12750406 PMC155042

[34] Katoh S Ishii N Nobumoto A Takeshita K Dai SY Shinonaga R Galectin-9 inhibits CD44-hyaluronan interaction and suppresses a murine model of allergic asthma. Am J Respir Crit Care Med (2007) 176:27–35. 10.1164/rccm.200608-1243OC 17446336

[35] Zhu L Zhuo L Kimata K Yamaguchi E Watanabe H Aronica MA Deficiency in the serum-derived hyaluronan-associated protein-hyaluronan complex enhances airway hyperresponsiveness in a murine model of asthma. Int Arch Allergy Immunol (2010) 153:223–33. 10.1159/000314362 20484920 PMC2945275

[36] Bhavsar P Ahmad T Adcock IM The role of histone deacetylases in asthma and allergic diseases. J Allergy Clin Immunol (2008) 121:580–4 10.1016/j.jaci.2007.12.1156 18234319

[37] Chen X Barozzi I Termanini A Prosperini E Recchiuti A Dalli J Requirement for the histone deacetylase Hdac3 for the inflammatory gene expression program in macrophages. Proc Natl Acad Sci U S A (2012) 109:2865–74. 10.1073/pnas.1121131109 22802645 PMC3479529

[38] Mullican SE Gaddis CA Alenghat T Nair MG Giacomin PR Everett LJ Histone deacetylase 3 is an epigenomic brake in macrophage alternative activation. Genes Dev (2011) 25:2480–8. 10.1101/gad.175950.111 22156208 PMC3243058

[39] Cao D Bromberg PA Samet JM. COX-2 expression induced by diesel particles involves chromatin modification and degradation of HDAC1. Am J Respir Cell Mol Biol (2007) 37:232–9. 10.1165/rcmb.2006-0449OC 17395887

[40] Adcock IM Ford P Ito K Barnes PJ Epigenetics and airways disease. Respir Res (2006) 7:21 10.1186/1465-9921-7-21 16460559 PMC1382219

[41] Kim Y Kim K Park D Lee E Lee H Lee YS Histone deacetylase 3 mediates allergic skin inflammation by regulating expression of MCP1 protein. J Biol Chem (2012) 287:25844–59. 10.1074/jbc.M112.348284 22679019 PMC3406670

[42] Kim Y Kim K Park D Lee E Lee H Lee YS DNA methyl transferase I acts as a negative regulator of allergic skin inflammation. Mol Immunol (2013) 53:1–14. 10.1016/j.molimm.2012.06.010 22784989

[43] Zhu H Shan L Schiller PW Mai A Peng T. Histone deacetylase-3 activation promotes tumor necrosis factor-α (TNF-α) expression in cardiomyocytes during lipopolysaccharide stimulation. J Biol Chem (2010) 285:9429–36. 10.1074/jbc.M109.071274 20097764 PMC2843192

[44] Park D Kim Y Kim H Kim K Lee YS Choe J Hyaluronic acid promotes angiogenesis by inducing RHAMM-TGFβ receptor interaction via CD44-PKCδ. Mol Cells (2012) 33:563–74. 10.1007/s10059-012-2294-1 22610405 PMC3887750

[45] Pasquinelli AE. MicroRNAs and their targets: recognition, regulation and an emerging reciprocal relationship. Nat Rev Genet (2012) 13:271–82. 10.1038/nrg3162 22411466

[46] Eom S Kim Y Park D Lee H Lee YS Choe J Histone deacetylase-3 mediates positive feedback relationship between anaphylaxis and tumor metastasis. J Biol Chem (2014) 289:12126–44. 10.1074/jbc.M113.521245 24619412 PMC4002117

[47] Zhou R Gong AY Chen D Miller RE Eischeid AN Chen XM. Histone deacetylases and NF-kB signaling coordinate expression of CX3CL1 in epithelial cells in response to microbial challenge by suppressing miR-424 and miR-503. PLoS One (2013) 8:e65153. 10.1371/journal.pone.0065153 23724129 PMC3665534

[48] Kaluza D Kroll J Gesierich S Manavski Y Boeckel JN Doebele C Histone deacetylase 9 promotes angiogenesis by targeting the antiangiogenic microRNA-17-92 cluster in endothelial cells. Arterioscler Thromb Vasc Biol (2013) 33:533–43. 10.1161/ATVBAHA.112.300415 23288173

[49] Chatterjee N Wang WL Conklin T Chittur S Tenniswood M. Histone deacetylase inhibitors modulate miRNA and mRNA expression, block metaphase, and induce apoptosis in inflammatory breast cancer cells. Cancer Biol Ther (2013) 14:658–71. 10.4161/cbt.25088 23792638 PMC3742495

[50] Chen CQ Chen CS Chen JJ Zhou LP Xu HL Jin WW Histone deacetylases inhibitor trichostatin A increases the expression of Dleu2/miR-15a/16-1 via HDAC3 in non-small cell lung cancer. Mol Cell Biochem (2013) 383:137–48. 10.1007/s11010-013-1762-z 23867991

[51] Polikepahad S Knight JM Naghavi AO Oplt T Creighton CJ Shaw C Proinflammatory role for let-7 microRNAs in experimental asthma. J Biol Chem (2010) 285:30139–49. 10.1074/jbc.M110.145698 20630862 PMC2943272

[52] Collison A Mattes J Plank M Foster PS. Inhibition of house dust mite-induced allergic airways disease by antagonism of microRNA-145 is comparable to glucocorticoid treatment. J Allergy Clin Immunol (2011) 128:160–7. 10.1016/j.jaci.2011.04.005 21571357

[53] Bourguignon LY Wong G Earle C Krueger K Spevak CC. Hyaluronan-CD44 interaction promotes c-Src-mediated twist signaling, microRNA-10b expression, and RhoA/RhoC up-regulation, leading to Rho-kinase-associated cytoskeleton activation and breast tumor cell invasion. J Biol Chem (2010) 285:36721–35. 10.1074/jbc.M110.162305 20843787 PMC2978601

[54] Henry JC Park JK Jiang J Kim JH Nagorney DM Roberts LR miR-199a-3p targets CD44 and reduces proliferation of CD44 positive hepatocellular carcinoma cell lines. Biochem Biophys Res Commun (2010) 403:120–5. 10.1016/j.bbrc.2010.10.130 21055388 PMC3039123

[55] Lou F Ma HN Xu L Chen M Zhu YB. Two polymorphisms of CD44 3’UTR weaken the binding of miRNAs and associate with naso-pharyngeal carcinoma in a Chinese population. Eur Rev Med Pharmacol Sci (2014) 18:2444–52. 25268088

[56] Lagendijk AK Goumans MJ Burkhard SB Bakkers J. MicroRNA-23 restricts cardiac valve formation by inhibiting Has2 and extracellular hyaluronic acid production. Circ Res (2011) 109:649–57. 10.1161/CIRCRESAHA.111.247635 21778427

[57] Röck K Tigges J Sass S Schütze A Florea AM Fender AC miR-23a-3p causes cellular senescence by targeting hyaluronan synthase 2: possible implication for skin aging. J Invest Dermatol (2015) 135:369–77. 10.1038/jid.2014.422 25264594

